# Quality of Life: Changes in Self-Perception in People with down Syndrome as a Result of Being Part of a Football/Soccer Team. Self-Reports and External Reports

**DOI:** 10.3390/brainsci11020226

**Published:** 2021-02-12

**Authors:** Rocío Camacho, Cristina Castejón-Riber, Francisco Requena, Julio Camacho, Begoña M. Escribano, Arturo Gallego, Roberto Espejo, Amaranta De Miguel-Rubio, Estrella I. Agüera

**Affiliations:** 1 Department of Cellular Biology, Physiology and Immunology, University of Cordoba, 14071 Cordoba, Spain; m92caagr@uco.es (R.C.); eabuendia@uco.es (J.C.); am1esdub@uco.es (B.M.E.); ba1agbue@uco.es (E.I.A.); 2 Department Artistic and Corporal Education, University of Cordoba, 14004 Cordoba, Spain; ccastejon@uco.es; 3 Department of Statistics, University of Cordoba, 14071 Cordoba, Spain; ma1gasea@uco.es (A.G.); ma1esmor@uco.es (R.E.); 4 Department of Nursing, Pharmacology and Physiotherapy, University of Cordoba, 14004 Cordoba, Spain; z42mirua@uco.es

**Keywords:** Down Syndrome, perception, quality of life, sport, age, gender, scale

## Abstract

The hypothesis posed was whether being part of a football/soccer team influenced the quality of life (QL) of the people who participated in it since their perception of themselves is enhanced by factors, such as self-determination, social inclusion, emotional well-being, physical well-being, material well-being, rights, personal development, and internal relationships. The objective was to evaluate the QL of people with Down Syndrome (DS) using their self-perception (*n* = 39) and the perception of the informants (family members, teachers) (*n* = 39). The KidsLife-Down Scale, with a few modifications, was used. In general, differences of opinion between the subgroups of participants with DS and informants showed that results were higher in terms of perception for participants in the DS subgroup. Scores for all variables were higher for those participants with DS who said they did engage in practicing competitive football/soccer. Although the perception of informants provides a great deal of information regarding the QL of participants with DS, participants with DS should also be involved in the evaluation process and their self-perceptions taken into account. It is not participating in a football team that causes the conclusions of the study, but training (which includes the friendly matches that are played), the cause correlated with the improvements detected in the athlete’s DS.

## 1. Introduction

The Cordoba Down Centre (CDC) is an NGO concerned with increasing the quality of life (QL) of people with Down Syndrome (DS) by promoting a healthy, autonomous, and independent lifestyle. 

QL occupies an important place in society because it is considered a way of measuring personal well-being. The need to assess quality of life has become a matter of great importance and practical utility for the development of good practices that, in accordance with the provisions of Spain’s Law 39/2006 dated 14 December 2006 on the Promotion of Personal Autonomy and Attention to Dependent Persons, has been included as an essential criterion in the accreditation process to guarantee the quality of centers, services, and the System for Autonomy and Attention to Dependent Persons (Resolution dated 2 December 2008 in Spain’s Official State Bulletin (BOE) published 17 December 2008). In the aforementioned resolution, these centers, services, and entities are required to present, among other things, documentation related to the user, including objectives, interdisciplinary work plan, interventions, and evaluation of results in terms of improvement in their quality of life. Currently, in Spain, according to the abovementioned law, instruments that allow the assessment of QL with sufficient guarantee of validity and reliability are indispensable.

QL has been defined as a series of objective biological, psychological, and social indicators that express a subjective evaluation of the degree to which life satisfaction has been achieved or the perceived level of personal well-being [1,2,3,4]. Schalock and Verdugo [5] proposed a model of QL defined as “the desired state of personal well-being from a multidimensional viewpoint, given that it includes both objective and subjective components and is also influenced by environmental factors and personal characteristics”. This model distinguishes eight essential aspects of quality of life and their corresponding indicators which are important for all people: social inclusion (participation, inclusion, and support), self-determination (goals, preferences, choice, and autonomy), emotional well-eing (satisfaction, absence of stress, motivation), physical well-being (nutrition, health, sport), material well-being (economic independence, technology, material support) rights (dignity, respect), personal development (adaptive behaviour, communication strategies, social skills), and interpersonal relations (friendship networks, autonomy). According to Claes et al. [6], the areas of emotional, physical, and material well-being, reflect the general well-being of the person; interpersonal relationships, social inclusion, and rights refer to social participation; personal development and self-determination express personal independence. Instruments to evaluate quality of life with a sufficient guarantee of validity and reliability are indispensable for dependent persons [7]. Given that interventions aimed at improving quality of life must be based on evidence, in Spain, the KidsLife Scale [8] was developed and validated for the evaluation of children and young adults with DS, using the model proposed by Schalok and Verdugo in 2003 [5].

The KidsLife Scale is intended to identify the person’s QL profile and provide evidence of validity and reliability for the implementation of evidence-based practices and the design of individual support plans. It provides standardized scores and percentiles for the eight core aspects of QL (emotional well-being, physical well-being, material well-being, personal development, interpersonal relationships, social inclusion, self-determination, and rights). It also allows the information obtained to be illustrated in a QL profile. This scale is aimed at childhood, adolescence, and youth.

The CDC includes a group of federated athletes who belong to the *Cordoba Football Club of LaLiga Genuine Santander, Spain*. Currently, in Spain, parallel to the Professional Football League, *LaLiga Genuine Santander* consists of a competitive national football league made up of people with intellectual disabilities. This league plays eight-a-side football in a single mixed category.

The objective of the present study was to evaluate the QL of people with DS at CDC using their self-perceptions and the perceptions of informants. To this end, we focused on: (1) Analysing the correlation of age in participants with DS and the informants with respect to aspects of QL; (2) Analysing differences in terms of gender in participants with DS and informants with respect to aspects of QL; (3) Verifying if there are differences in aspects of QL between those who practice competitive sport and those who do not, according to the self-perceptions of participants with DS and the opinions of the informants, and finally (4) Evaluating differences of opinion with regard to the aspects of QL between groups (people with DS and informants). 

With this study, we wanted to emphasize that, in spite of the fact that the perception of informants provides a great deal of information regarding the QL of participants with DS, participants with DS should also be involved in the evaluation process and their self-perceptions taken into account.

Scale hypotheses for people with Down syndrome was:

Gender, age and being part of a football/soccer team improve the quality of life for people with DS. The perception of your QL should coincide with the perception thereof on the part of informants.

Scale hypothesis for informants was:

Gender, age and being part of a football/soccer team contribute to improve the quality of life for people with DS. The perception of QL should coincide with the perception on the part of people with DS.

## 2. Materials and Methods

### 2.1. Participants

A total of 78 people participated in the study, 39 with DS who were users of CDC, with an age between 21–40 years (29 ± 3) (men *n* = 24; women *n* = 15; athletes *n* = 9, non-athletes *n* = 30) and 39 informants. Here, “athletes” refers to the federated footballers belonging to a football/soccer team (the *Cordoba Football Club of LaLiga Genuine Santander, Spain)*; “non-athletes” were non-federated and did not participate in that team. 

The informants (family members, teachers) needed to know the participant well for at least six months and have the opportunity to observe them in different environments for prolonged periods. The relationship of the informants with the person evaluated was 34 parents and 5 teachers (87.17% parents and 12.82% teachers). The informants for athletes were 8 parents and 1 teacher (88.89% parents and 11.11% teachers); informants for the group of non-athletes were 26 parents and 4 teachers.

The sociodemographic data used in forming the work groups were collected by each informant before proceeding with evaluation: age, gender, place of birth, percentage of recognized disability, intellectual disability in terms of adaptive behavior (conceptual, social and practical skills), recognized level of dependence (moderate, severe, high dependence), other assessed conditions (physical, auditory or visual disability, obesity, etc.). All participants with DS were Spanish, Caucasian, with a medium-high socioeconomic level. The percentage of recognized disability ranged from 73–75%.

Both athletes and non-athletes with DS participated in two regular sessions of Physical Education at CDC in which basic movement patterns were practiced to resolve motor difficulties in daily life using various circuits and posts (jumping, throwing, coordination, and balance) as well as improving basic physical qualities: strength, speed, stamina, and range of movement. Finally, various sports were practiced (basketball, football, etc.), which included the use of balls in games modified and adapted to the participants’ different levels of ability.

Outside CDC, athletes also took part in two 90 min training sessions a week under the supervision of a coach. Each session consisted of a warm-up period, the main session, and a cool-down period:

(a) Warm-ups were divided into general warm-ups, in which the participants activated the neuromuscular system with group games, followed by specific warm-ups for which the goalkeepers were separated from the field players and specific motor activities were practiced.

(b) During the main session, balls were used, and the specific technical and tactical moves of football were practiced (control, passing, shooting, etc.) Strategic roles were distributed for each side (offense player with ball, offense player without ball, defensive player, goalkeeper). Later, real play situations were practiced in short games, changing the rules to meet the objective established for each session.

(c) During cool-down, the participants did stretches.

This type of training is more demanding of motor skills than the routine sessions at CDC.

All participants with DS lived with their families. 

### 2.2. Method

Previous to the study, the objective was presented to the directors of CDC to obtain their ethical approval and the consent of those involved or their family members. The authors of this study declare that, based on the Helsinki Declaration, they have taken into account the basic principle of respect for the individual, his/her right to self-determination, and to make decisions once clearly informed of the pros and cons, risks and benefits of participating in this research study [9]. The study was carried out respecting the ethical standards of the CDC committee. Once written consent was obtained, a meeting was held with the participants with DS and informants to discuss rules of application and proper use, as well as to warn informants not to influence the responses of participants with DS, though they could clarify points as needed. Participants with DS were told they could request clarification of anything they did not understand. 

The first author of this study then sent the QL scale to CDC, who distributed it to parents, teachers, and coordinators (a printed version and via email). The scales were gathered by a CDC liaison. Once filled out, the researchers compiled the answers in a database and carried out the pertinent statistical analyses. During the process of administering the scale, no personal data were compiled that might identify the person under evaluation. Instead, identification codes were used (such as pseudonyms) that were unknown to the researchers to protect confidentiality, in accordance with Spain’s Organic Law 3/2018 on the protection of personal data and guarantee of digital rights. These identification codes allowed the results of the evaluations to be returned to CDC to be used in later interventions with the participants [10].

Once ethical approval and acceptance for participation in the study were obtained, the researchers did not select the participants; rather, they voluntarily agreed to participate. They were not given any incentives.

### 2.3. Instrument 

A modified version of The KidsLife-Down scale [8] was used to evaluate QL. Participants with DS responded with one of two options (dichotomy) and informants with a Likert scale. All 78 participants (DS and informants) answered the scale.

The scale consisted of items divided into eight aspects of QL (self-determination, rights, emotional well-being, material well-being, physical well-being, social inclusion, interpersonal relationships, and personal development) [11]. This scale provides standardized scores and percentiles for the eight aspects, as well as a QL profile report.

There were two versions of the scale used: (a) a self-report filled out by participants with DS, with two options (yes/no) and (b) an external report filled out by informants using a Likert scale with four frequency options (never, sometimes, often, always) [12]. The questions asked of informants were the same as those answered by participants with DS, but in the third person.

Direct scores for each aspect of QL were the sum of the scores for the items in each section. The direct scores were then converted to standard scores (M = 10; SD = 3) following the 15-to-21-year age range provided by the scale. The total standard score was obtained by adding up the standard scores for the eight aspects, which was then converted to the standard composite score or Quality of Life Index (QLI) (M = 100; SD = 15) [8], taking into account the aforementioned range.

High scores for the various aspects of QL and QLI indicate a high level of functioning for the person in a given area, greater QL, and personal well-being. All scores can be shown in a graph of the QL profile [5].

At the time of writing the survey questions, we attempted to avoid any cognitive bias in the two groups of respondents so as to obtain honest information. For people with DS, the questions were written using personal, direct language (Table 1). To facilitate their responses, the dichotomous (Y/N) type of response was chosen. Questions that were considered more complex were stated in a simpler way or using colloquial language. It was found that the formulation of the questions did not influence the answers, nor did it induce inaccuracies in relation to the information collected [13]. In addition, the sample bias was taken into account to ultimately obtain reliable information of good quality (The requirements of the respondents to fulfill the objectives of the work were clearly defined [13]. There were two different responses (dichotomy and Likert scale), and these were scored so as to obtain (M = 10) for all of them.

### 2.4. Method of Scale Validation

The scale used was validated by Gómez et al. [8]. To validate the modifications introduced, the validation process was carried out by a team of professional experts belonging to the CDC’s board of directors. This committee did not participate as informants. The earliest version of the questionnaire was sent to CDC, who reviewed the possible errors in formulating the questions. They provided feedback that served to reformulate the questions in an appropriate way to avoid confusion among the people surveyed.

Feedback focused mainly on the following issues: wording of questions, vocabulary related to the context of CDC, elimination of ambiguous questions in favor of more specific ones, removal of terminology that could be interpreted as being patronizing or offensive, and benefits of some questions regarding the logic of the questionnaire.

The revision of the questionnaire was carried out with an in-depth analysis of all contributions so that it included those that could be considered adequate to allow for the drafting of a definitive model. The improved version was again forwarded to CDC. The questionnaire was considered non-offensive, comprehensible, and suitable for participants.

To validate the reliability of the questionnaire, verify and confirm the matter under investigation, Cronbach’s alpha consistency coefficient was used [14,15,16].

### 2.5. Statistical Analysis 

Normality compliance was tested for each group using the variables of gender, age, and football (to practice competitive football/soccer or not) via the Kolmogorov–Smirnov test. The aforesaid hypothesis was not met for all variables studied (*p* < 0.001 in all cases); therefore, non-parametric or free distribution tests were applied, specifically Spearman’s Rho (rank-order correlations) and Mann–Whitney’s *U*-tests. The SPSS program (v25; IBM, Armonk, NY, USA) was used for all statistical analyses of data.

## 3. Results

The psychometric properties of the scale were satisfactory. The questionnaire answered by participants with DS obtained a Chronbach alpha coefficient of 0.6, and that answered by the informants obtained a coefficient of 0.87. 

The randomness of the sample was verified with the Runs test (Wald–Wolfowitz), obtaining *Z* < 0.001, *p* > 0.05, which showed that it was random.

### 3.1. Intellectual Disability and Other Conditions 

Participants with DS presented a predominant moderately high level of intellectual disability (in terms of adaptive behavior) of 50%: in detail, 56% in conceptual skills, 51.3% in social skills, and 54% in practical skills. The percentage of recognized disability ranged from 73–75%. Other conditions evaluated showed that 25.5% had physical disabilities, 44% obesity, 18.3% sensorial disability, 6.3% had serious health problems, and 4.9% had sleep disorders. Table 2 clarifies the descriptive statistics (%) for the level of intellectual disability (in terms of adaptive behavior) and the level of recognized dependence. An analysis of variance (ANOVA) was used to test the hypothesis that the means of athletes and non-athletes were equal. The *p*-value obtained for all variables was greater than the level of significance; therefore, there were no significant differences in the results obtained, and the groups did not show a priori differences in intellectual disability and level of dependence.

### 3.2. Age

In the analysis of age correlation for both participants with DS and informants with respect to quality of life, the Kolmogorov–Smirnov test showed that normality compliance was not achieved. Therefore, Spearman’s Rho (rank–order correlations) was used for the subsample of participants with DS (*n* = 39) as well as the subsample of informants (*n* = 39), using the age scale and all aspects of QL implied in the study (Table 3).

Results for the subsample of participants with DS indicated a single statistically significant correlation (*r* = −0.353; *p* = 0.027) with moderate magnitude and negative meaning with respect to the physical well-being variable. No other significant relationship was detected for the remaining variables, including QLI. However, no statistically significant relationship was detected between the age of participants with DS and the opinions of informants in terms of any aspect of the study.

### 3.3. Differences in Terms of Gender

In the analysis of differences in terms of gender, with respect to the aspects studied and QLI of participants with DS and informants, the Kolmogorov–Smirnov test showed that normality compliance was not achieved. Therefore, to contrast the differences between both groups (participants with DS and informants), non-parametric testing was applied, equivalent to Student *t-*test for independent groups, Mann–Whitney *U*-tests (Table 4).

Results for self-perception of participants with DS indicated significant differences for the emotional well-being variables (Z = −2.29; *p* = 0.022), material well-being (Z = −2.29; *p* = 0.022), and personal development (Z = −2.20; *p* = 0.028). For these three variables, results were higher for men. No statistically significant difference was detected for the remaining variables nor for QLI (Table 4).

In the second place, with regard to informants, statistically significant differences were detected between genders for participants with DS for the variables social inclusion (Z = −2.49; *p* = 0.013), emotional well-being (Z = −2.29; *p* = 0.022), physical well-being (Z = −2.45; *p* = 0.014), material well-being (Z = −3.88; *p* < 0.001), and QLI (Z = −2.84; *p* = 0.004). For all five variables, results were higher for men. No statistically significant difference was detected for the remaining variables (Table 4).

Therefore, the opinions of participants with DS and informants coincided with respect to emotional well-being and material well-being.

### 3.4. Differences between Variables in the Study and QLI between Athletes and No Athletes 

To verify if there were differences between variables in the study and QLI between athletes and no athletes, according to the self-perceptions of participants with DS and in the opinion of informants, the non-parametric Mann–Whitney *U*-test was again applied. Results are shown in Table 5.

The opinion of participants with DS showed statistically significant differences between the group with DS who practiced competitive football/soccer and those who did not. These results for all variables, including QLI, were higher for those participants who said they practiced competitive football/soccer (in all cases, *p* < 0.001; Table 5).

In the second place, with reference to informant opinion, no statistically significant difference was detected for any of the variables as regards the practice or not of competitive football/soccer on the part of participants with DS.

### 3.5. Differences of Opinion between Participants with DS and Informants

Differences of opinion were also evaluated concerning the variables studied and QLI between participants with DS and informants. A new series of Mann–Whitney *U*-tests was applied to contrast differences between both groups of participants, those with DS and informants. Results are shown in Table 6. 

In this case, the results showed statistically significant differences (Table 4) between participant groups with reference to social inclusion (Z = −2.89; *p* = 0.004), self-determination (Z = −4.25; *p* = 0.001), material well-being (Z = −2.88; *p* = 0.004), personal development (Z = −2.39; *p* = 0.017), and finally QLI (Z = −3.27; *p* = 0.001). In all aspects mentioned in QLI, results were higher in terms of perception for participants with DS.

## 4. Discussion

This is a unique study. The Genuine League began in 2017, and this is the first time that a Quality of Life survey has been carried out among highly competitive athletes with these characteristics. People with DS are sedentary by definition [17].

Evaluation of QL for CDC users was carried out using the modified KidsLife Scale [8]. The KidsLife-Down Scale is a scale for informants. In this study, the authors have included another scale aimed at people with DS, which is one of the main novelties of this study. This pioneer scale allowed the compilation of in-depth distinctions for those who responded to the questionnaire from two points of view: that of those with DS and that of informants. Though the number of participants in the study was relatively small, important results were found.

In the scientific literature, there are authors who believe that for a scale to be reliable, it must obtain a Cronbach’s coefficient of between 0.65–7 [14,15,16]. The authors of this research have established a new level of reliability at 0.6 for the scale when answered by people with DS. It must be borne in mind that sociodemographic data showed that the level of intellectual disability (in terms of adaptive capacity) of participants was moderate. Scientific literature shows that persons with DS have certain limitations associated with cognitive capacity, which show up in adaptive capacity (conceptual, social, and practical skills) [18,19]. Adaptive skills coincide with the level of intelligence, which implies that there are no severe limitations in functionality, as long as the degree of intellectual disability is not profound or severe [20,21]. In adulthood, a person is expected to be able to deal with the demands of daily life and, in turn, those demands corresponding to relationships with family, friends, and CDC staff. However, people with DS present behavior that is sometimes classified as atypical [22].

It has been verified that the sample of participants was homogeneous in terms of the level of intellectual disability (as regards adaptive behavior) and the recognized level of dependence since no significant differences were found in these variables. Adaptive behavior is the set of conceptual and social skills and practices that are learned and used by people in their daily lives [23]. Individual functioning will depend on personal characteristics of intellectual ability, adaptive behavior, health, participation in social life, and the context within which the person functions. In addition, it will depend on the support provided [24].

Perception of the aspects on which QL is based varies with reference to each specific person’s QL. Therefore, significant differences were found when participants evaluated their own QL versus when informants gave opinions regarding third parties, particularly those with DS, which coincides with studies carried out by [25,26]. This confirmed that, in line with CDC’s purpose, this population’s QL must be fomented. As proposed by Shalock and Verdugo [11], QL is composed of the same aspects and indicators, having the same degree of importance, for all people [26]. However, the results of the present study do not coincide with the studies of QL carried out by Córdoba et al., [27]; Bagnato et al., [28]; Vega et al., [29]. Consequently, the importance of having two viewpoints must be reflected to evaluate the QL of these persons properly. Bagnato et al. [28] attributed this to the fact that informants and people with intellectual disabilities share an extensive daily schedule of interaction, and, therefore, teachers develop important knowledge about the participants with DS. They consider that the absence of significant differences between the informants’ responses confirms the usefulness and reliability of the scale both with primary informants and with relatives, to assess the perception of life satisfaction. However, in our view, one of the main contributions of this work is that it reflects the importance of having two points of view to assess the QL of people with DS properly.

Regarding age, on the one hand, participants with DS perceived that with respect to all aspects of QL, physical well-being diminishes as age increases. This perception on the part of participants with DS may be due to the fact that adults in this population suffer from “accelerated aging”, which implies experiencing certain physical conditions common among people of advanced age in the general population. The reason for this is not fully understood but is related in large part to the genes of Chromosome 21 associated with the aging process [10]. Premature aging means that in society, we find people with DS who, although they do not meet the age criteria to be considered elderly, already present geriatric syndromes years in advance [30]. As a consequence, adults with DS have lower longevity compared to the general population of people with intellectual disabilities [31]. Perhaps they perceive their physical well-being in a negative way due to physiological changes, which can increase the risk of chronic degenerative diseases [32] and, consequently, important limitations on activity [33]. In any case, the aging process as such is dynamic and variable depending on the individual context since it is continuously influenced by external and internal agents and multiple factors, such as lifestyle [31].

On the other hand, informants did not share this perception. Preoccupation with physical well-being, and health, in particular, is an outstanding and determining indicator of QL for aging persons with intellectual disabilities. The explanation can be found in the fact that the subject’s perception is radically modified when he or she presents serious health problems [34]. Perhaps the informants did not take into account the associated pathologies suffered by users with physical disabilities: obesity, sensorial disability, serious health problems, or sleep disorders. In this study, as in Aja et al. [35] and Badía et al., [12], it was shown that age had no significant relationship to QL. Aja et al. [35] found a significant inverse association between age and the aspects of social inclusion and personal development. Thus, it seems that for people over 35 years of age with intellectual disabilities, it is important to include programs that promote personal development and favour social inclusion. However, these results differ from those obtained by other researchers [36,37], who found that there was a significant correlation between quality of life and age. 

In the present study, statistically significant differences were shown with respect to gender as perceived by participants with DS for the variables of emotional well-being (personal satisfaction, motivation, absence of stress), material well-being, and personal development (adaptive behavior, competence, social skills, and development of communication); these were higher in men than in women. These results coincide with [38,39,40,41], which also pointed out that men had higher emotional well-being than women. However, we differ from the foregoing authors who stated that women have a lower quality of life than men since in this research, we found no significant differences in QLI. Significant differences with respect to emotional well-being may be due to the fact that women are more expressive of emotion and more aware of life events [42]. Emotional well-being is a balance between feelings, desires, and emotions. A great difference is often found between emotional age, cognitive development, and chronological age. Infantilizing people with DS puts them at risk and marginalizes them [43]. Differences in material well-being can be attributed to the fact that women attain greater job placement, as well as being more protected by family members than men; they exchange free time for family support [44]. Greater personal development in men may indicate that they have learned better skills and habits that make them more competent [11].

Informants showed significant gender-related differences in social inclusion, emotional well-being, physical well-being, material well-being, and QLI, with men being favored; this coincided with participants with DS with respect to emotional and material well-being. The differences and biological peculiarities of men and women were taken into account, as well as their interaction with gender-related social factors, such as identity, roles, responsibilities, and strengths, which are reflected in emotional and material health as well as social inclusion for both sexes [45,46]. Positive emotions have a very beneficial effect on the well-being of all people [47]. They are extremely important for people with intellectual disabilities, as they can help them deal decisively with the barriers they may encounter in society. On the other hand, the family is a fundamental agent that must generate contexts in which future plans can be made, especially regarding employment, and in which this population is included in a normalized adult life [48].

In spite of playing in a single mixed category, by chance *LaLiga Genuine Santander Football League* includes no female users of CDC. In the opinion of CDC, their users’ participation in training and intervention in practice/friendly football matches not only contributes to the stimulation of motor skills of those members with DS but also includes those health, cultural, and social aspects that accompany sport and reinforce a healthy lifestyle, values, and attitudes in participants. Besides, it is a way to optimize social skills as well as emotional, psychological, and physical health [49].

No agreement was verified in terms of the perceptions of participants with DS and informants regarding aspects of QL between those who practiced competitive football/soccer and those who did not. While informants did not show significant differences in any of the aspects evaluated in athletes and non-athletes, the results obtained from participants with DS showed statistically significant differences for all variables, including QLI. All variables obtained higher values for participants in competitive football/soccer versus those who did not participate in that sport. This result was very striking, as we had thought that informants would perceive an improvement in QL as a result of being part of a football/soccer team since team sports are an activity that increases the majority of variables contributing to quality of life [49], providing an opportunity to interact and share with others and, therefore, integrate into society [50]. As shown in other studies [51,52,53,54,55], sport foments mutual awareness and cooperation, making it an ideal way to create social capital. In particular, football is a socio-motor sport of cooperation/opposition, which within the context of attack/defense represents a form of social activity that demands high levels of coordination as well as encouraging communication between teammates (passes, support, etc.) and opponents (scores, charges, intercepting the ball) [56]. We agree with other authors [49,57] that sport foments interpersonal relationships, social inclusion, self-determination, and quality of life. Competitive team sports are characterized by intense social and physical contact. The context of sport represents society’s virtues and defects on a large scale, which may serve to reflect the socialization of the athlete in the relationships formed with teammates, coaches, family, and peers [58], as well as improving QL [59].

Finally, upon comparing QL variables between participants with DS and informants, it was clear that the former had a higher perception compared to the latter. The results obtained coincide with those of [41], who concluded that the perception of people with intellectual disabilities was higher than the perception of the professionals in charge of them. However, in 1999, Stancliffe [60] found no significant differences between different informants. In 2017, Flórez [61] stated that the immense majority of people with DS are happy with their lives, appearance, and personality. These significant differences in which the opinions of informants do not coincide with those of participants with DS are the variables of self-determination, material well-being, personal development, and QLI. The informants acknowledged the difficulties people in their care have in taking responsibility for themselves, participating independently in their environment, becoming economically independent, and making autonomous decisions; this is in line with the findings of other researchers who indicated that the disabled perceive themselves as less self-determined than their peers without disabilities [38,41,62,63]. We consider that the difference in perception of QL on the part of people with DS and informants reflects the fact that the two groups do not share the same satisfactions and concerns.

The value of quality of life evaluated by participants with DS may generate debate regarding the reliability and validity of their responses [64]; however, knowing their opinions is necessary since quality of life has a very personal (subjective) side [65]. Some authors agree that subjective factors must be evaluated from the viewpoint of those with intellectual disabilities; to this end, abstract questions must be avoided for the members of this population to understand [66,67]. According to [68], there are significant differences between the perceptions of the disabled and those of informants.

There are some limitations to the present study; one of these is the sample size. Another is the fact that in spite of the league being mixed, no women users of CDC participate in the *Cordoba Football Club of LaLiga Genuine Santander*; therefore, gender-based comparisons could not be made. It must be borne in mind that the impact of the practice of sport on different aspects of quality of life may be modulated by environmental or intrapersonal factors: age, sex, social skills, adaptive behavior, and degree of disability, as well as the kind of sport and access to other leisure activities [69,70]. Finally, other variables were not included, such as the need for support, living in assisted living facilities, or inclusion in a job placement program.

We suggest that future research should broaden the scale to include other Spanish football/soccer teams of people with intellectual disabilities to contrast the opinions of participants with DS and informants regarding football practice.

## 5. Conclusions

Participants with DS perceived that increased age is associated with decreased wellbeing. However, informants did not share this perception. The opinions of participants with DS and informants regarding gender showed significant differences, coinciding only in terms of emotional and material well-being.

Scores for all variables were higher for those participants who said they engaged in practicing competitive football. However, informants did not perceive that QL depended on the participants with DS practicing football or not.

In general, differences of opinion between the subgroups of participants with DS and informants showed that results were higher in terms of perception for participants in the DS subgroup.

In spite of the fact that the perception of informants provides a great deal of information regarding the QL of participants with DS, the latter should be involved in the evaluation process and their self-perceptions taken into account. Therefore, self-reporting is a necessary tool for this population to be able to evaluate their own QL; avoiding abstract questions is fundamental to aid understanding. The ideal is a combination of self-reporting with reports by informants.

It was not participating in the league that causes the conclusions of the study, but training (which includes the friendly matches that are played), the cause correlated with the improvements detected in the athlete’s DS.

## Figures and Tables

**Table 1:** Examples of questions for the informants and people with Down Syndrome (DS) view.

Informants	People with DS
Take the recommended amount of food and fluids to maintain good health.	Do you eat everything your parents or the Association give to you?
Has he/she adequate hygiene (e.g., teeth, hair, nails, body) and personal image (e.g., clothing and accessories appropriate for their age and for the occasion).	Do you wash your teeth, hair, nails, and body? Do you wear the clothes you like?
Performs activities and physical exercises appropriate to their characteristics and needs.	Do you practice physical activity in any sport outside of the Association: football/soccer, swimming, basketball…?
Does he/she have a preventive health plan (e.g., regular tests, specialist reviews)	Do you go to the doctor even if you are not sick for a check-up (e.g., blood test)?

**Table 2:** Descriptive statistics (%) of the level of intellectual disability and level of dependency recognized of participants with DS (*n* = 39).

			Category			
		Total	Athletes	No Athletes		
Variables	Level	%	%	%	F	*p*
Conceptual skills	Mi	28.2	55.6	20		
	Mo	56.4	44.4	60	5.55	>0.01
	Se	15.4	0	20		
Social skills	Mi	38.5	66,7	30		
	Mo	51.3	33,3	56.7	4.52	>0.01
	Se	10.3	0	13.3		
Practical skills	Mi	35.9	55.6	30		
	Mo	56.4	33.3	63.3	0.84	>0.01
	Se	7.7	11.1	6.7		
Recognized level of dependency	Mo	20.5	22.2	20		
	Se	12.8	22.2	10	0.87	>0.01
	Hd	10.3	22.2	6.7		

Note: Mi: Mild; Mo: Moderate; Se: Severe; Hd: High dependency.

**Table 3:** Spearman’s Rho correlations between age of participants with DS (*n* = 39) and self-perception with respect to the aspects of the study, and the correlation of these ages with informant perceptions (*n* = 39).

	Age			
	DS		Informants	
Dependent variables	*r*	*p*	*r*	*p*
Social inclusion	−0.44	−0.792	−0.057	−0.728
Auto-determination	−0.212	−0.196	−0.081	−0.622
Emotional well-being	−0.246	−0.131	−0.093	−0.572
Physical well-being	−0.353	−0.027	−0.012	−0.942
Material well-being	−0.062	−0.708	−0.120	−0.474
Rights	−0.083	−0.614	−0.114	−0.490
Interpersonal relationship	−0.135	−0.411	−0.011	−0.946
Pesonal development	−0.219	−0.181	−0.074	−0.656
Quality life index	−0.194	−0.237	−0.204	−0.212

**Table 4:** Mann–Whitney U tests for independent variables of the study with respect to gender for the subgroup of participants with DS (men *n* = 24; women *n* = 15) concerning self-perception and perception of informants (*n* = 39).

		DS Participants								Informants							
Dependent Variable	Gender (DS)	Mean	SD	Min	Max	Average Range	U_MW_	Z	*p*	Mean	SD	Min	Max	Average Range	U_MW_	Z	*p*
SI	M	5.58	1.586	4	8	21.85	135.50	−1.350	0.177	4.83	1.239	3	7	23.23	102.50	−2.488	0.013
	F	4.73	0.704	4	6	17.03				3.93	0.961	3	6	14.83			
AU	M	5.71	1.517	3	8	22.69	115.50	−1.899	0.058	4.13	0.900	3	5	22.50	120.00	−1.841	0.066
	F	4.73	1.387	3	8	15.70				3.60	0.632	3	5	16.00			
EW	M	5.00	1.142	4	7	23.06	106.50	−2.294	0.022	5.08	1.412	3	7	23.21	103.00	−2.289	0.022
	F	4.13	0.834	3	6	15.10				4.07	0.884	3	6	14.87			
PW	M	9.08	0.717	8	10	21.98	132.50	−1.463	0.143	8.88	0.947	8	10	23.25	102.00	−2.446	0.014
	F	8.53	1.125	7	10	16.83				8.00	0.845	6	9	14.80			
MW	M	6.96	1.197	5	9	23.15	104.50	−2.294	0.022	6.43	1.273	4	8	24.98	46.50	−3.875	<0.001
	F	6.07	1.033	5	8	14.97				4.73	0.704	4	6	11.10			
R	M	4.79	1.817	3	8	20.50	168.00	−0.357	0.721	4.88	1.676	3	8	20.42	170.00	−.304	0.761
	F	4.27	0.961	3	6	19.20				4.40	0.986	3	7	19.33			
IR	M	5.46	1.285	4	7	20.77	161.50	−0.567	0.571	5.21	1.141	4	7	21.60	141.50	−1.184	0.236
	F	5.20	1.207	4	7	18.77				4.73	0.799	4	7	17.43			
PD	M	5.42	1.248	4	7	23.06	106.50	−2.203	0.028	4.46	0.658	3	5	21.60	141.50	−1.229	0.219
	F	4.47	0.915	3	6	15.10				4.20	0.676	3	5	17.43			
QLI	M	72.71	8.800	63	86	21.75	138.00	−1.238	0.216	68.96	7.369	62	80	23.44	97.50	−2.841	0.004
	F	67.53	3.701	63	73	17.20				63.00	0.000	63	63	14.50			

NOTE: M: Male; F: Female; DS: People with Down syndrome; SI: Social inclusion; AU: Auto-determination; EW Emotional well-being; PW: Physical well-being; MW: Material well-being; R: Rights; IR: Interpersonal relationship; PD: Personal development; QLI: Quality life index.

**Table 5:** Mann–Whitney *U*-tests for dependent variables with respect to practicing competitive football on the part of participants with DS (Yes, *n* = 9; No, *n* = 30) according to the opinions of the subgroup with DS and informant perceptions (*n* = 39).

		DS Participants								Informants							
Dependent Variable	Football (SD)	Mean	SD	Min	Max	Average Range	U_MW_	Z	*p*	Mean	SD	Min	Max	Average Range	U_MW_	Z	*p*
SI	Y	7.44	0.726	6	8	34.89	1.00	−4.695	<0.001	4.56	1.130	3	6	20.94	126.50	−0.315	0.781
	N	4.60	0.621	4	6	15.53				4.47	1.252	3	7	19.72			
AU	Y	7.33	0.707	6	8	33.89	10.00	−4.249	<0.001	4.22	0.833	3	5	23.83	100.50	−1.222	0.255
	N	4.73	1.143	3	8	15.83				3.83	0.834	3	5	18.85			
EW	Y	6.33	0.500	6	7	34.67	3.00	−4.757	<0.001	4.78	1.481	3	7	20.56	130.00	−0.172	0.883
	N	4.17	0.648	3	6	15.60				4.67	1.295	3	7	19.83			
PW	Y	9.78	0.441	9	10	31.39	32.50	−3.645	<0.001	8.67	1.000	8	10	20.67	129.00	−0.217	0.857
	N	8.60	0.855	7	10	16.58				8.50	1.009	6	10	19.80			
MW	Y	8.33	0.500	8	9	34.33	6.00	−4.525	<0.001	6.25	1.389	4	8	23.69	86.50	−1.235	0.235
	N	6.10	0.803	5	8	15.70				5.63	1.351	4	8	18.38			
R	Y	6.89	1.054	5	8	34.44	5.00	−4.465	<0.001	4.67	1.581	3	7	19.17	127.50	−0.263	0.806
	N	3.90	0.845	3	6	15.67				4.70	1.442	3	8	20.25			
IR	Y	7.00	0.000	7	7	33.00	18.00	−4.138	<0.001	5.33	1.323	4	7	22.17	115.50	−0.692	0.522
	N	4.87	0.973	4	7	16.10				4.93	0.944	4	7	19.35			
PD	Y	6.67	0.500	6	7	33.83	10.50	−4.308	<0.001	4.33	0.866	3	5	20.39	131.50	−0.129	0.909
	N	4.57	0.898	3	7	15.85				4.37	0.615	3	5	19.88			
QLI	Y	83.33	1.500	81	86	35.00	0.00	−4.594	<0.001	67.78	7.225	62	79	20.83	127.50	−0.298	0.806
	N	66.93	3.423	63	73	15.50				66.33	6.283	63	80	19.75			

NOTE: Y: Practicing competitive football/soccer (Yes); N: Not Practicing competitive football/soccer (No); DS: People with Down syndrome; SI: social inclusion; AU: auto-determination; EW Emotional well-being; PW: Physical well-being; MW: Material well-being; R: Rights; IR: Interpersonal relationship; PD: Personal development; QLI: Quality life index.

**Table 6:** Mann–Whitney *U*-tests for dependent variables studied with respect to groups (participants with DS, *n* = 39; Informants, *n* = 39).

Dependent Variable	Group	Mean	SD	Min	Max	Average Range	U_MW_	Z	*p*
Social inclusion	DS	5.26	1.371	4	8	46.50	487.50	−2.889	0.004
	I	4.49	1.211	3	7	32.50			
Auto-determination	DS	5.33	1.528	3	8	50.08	348.00	−4.246	<0.001
	I	3.92	0.839	3	5	28.92			
Emotional well-being	DS	4.67	1.108	3	7	39.42	757.50	−0.031	0.975
	I	4.69	1.321	3	7	39.58			
Physical well-being	DS	8.87	0.923	7	10	43.62	600.00	−1.681	0.093
	I	8.54	0.996	6	10	35.38			
Material well-being	DS	6.62	1.206	5	9	46.03	467.00	−2.880	0.004
	I	5.76	1.364	4	8	31.79			
Rights	DS	4.59	1.551	3	8	38.44	719.00	−0.430	0.667
	I	4.69	1.454	3	8	40.56			
Interpersonal relationship	DS	5.36	1.246	4	7	42.05	661.00	−1.053	0.292
	I	5.03	1.038	4	7	36.95			
Personal development	DS	5.05	1.213	3	7	45.28	535.00	−2.389	0.017
	I	4.36	0.668	3	5	33.72			
Quality life index	DS	70.72	7.643	63	86	47.41	452.00	−3.268	0.001
	I	66.67	6.441	62	80	31.59			

NOTE: 1: People with Down syndrome; 2: Informants.

## Data Availability

Due to ethical, legal or privacy issues are present, data should not be shared.
